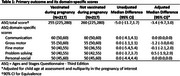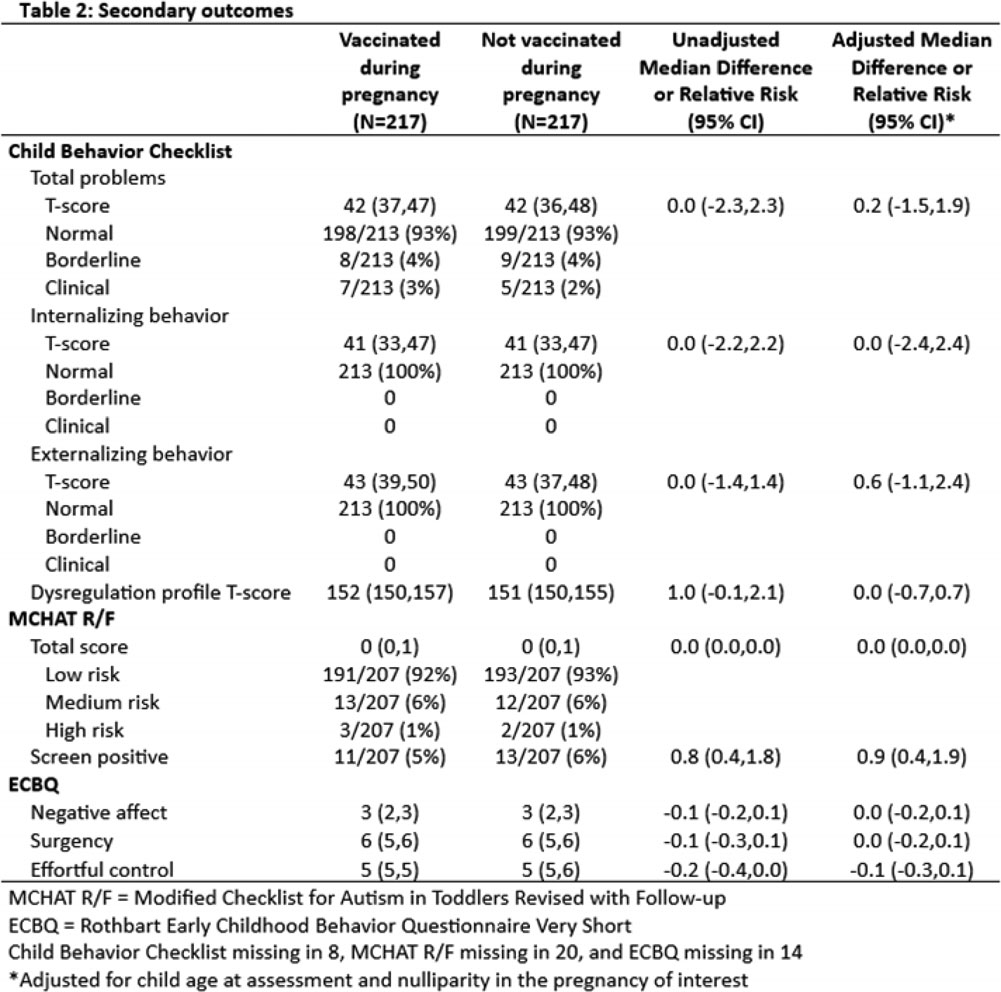# Oral Plenary Session 1: Wednesday, February 11, 2026 8:00 AM – 10:00 AM

**DOI:** 10.1002/pmf2.70150

**Published:** 2026-02-09

**8:00 AM – 8:15 AM**

## 1 | A Randomized Controlled Trial of Baby2Home: A Postpartum Digital Mental Health Intervention

Emily S. Miller^1^; Jacqueline Gollan^2^; Lutfiyya N. Muhammad^3^; Kathleen O'Sullivan^2^; Dinah Williams^4^; Malika D. Shah^3^; Lynn M. Yee^3^; Siyuan Dong^3^; Young S. Lee^3^; Craig F. Garfield^2^

^1^Women & Infants Hospital of Rhode Island / Warren Alpert Medical School of Brown University, Providence, RI; ^2^Northwestern University Feinberg School of Medicine, Evanston, IL; ^3^Northwestern University Feinberg School of Medicine, Chicago, IL; ^4^Women & Infants Hospital of Rhode Island, Providence, RI

**Objective**: Mental health conditions are among the most common perinatal complications and contribute significantly to maternal morbidity and mortality in the US. Access to timely, effective mental health care remains limited. We aimed to evaluate whether Baby2Home (B2H), a collaborative care model‐based digital health intervention co‐designed with new parents, improves mental health, relationship health, and self‐efficacy outcomes over the first year postpartum.

**Study Design**: We conducted a randomized controlled trial of B2H vs. usual care among postpartum primiparas with term infants recruited from two large birthing hospitals. B2H is a digital health tool that includes infant care trackers designed to boost parenting confidence and communication, individualized anticipatory education, mental health self‐management tools, and a care manager for on‐demand mental health and problem‐solving support. Patient‐reported outcomes, including stress (PSS), depression (PHQ9), anxiety (GAD7), global health (PROMIS‐GH), relationship satisfaction (RDAS), and self‐efficacy (PROMIS‐SE), were collected electronically at 1, 2, 4, 6, and 12 months postpartum. Analyses followed intention‐to‐treat principles and used linear mixed‐effects models to estimate between‐group differences over time.

**Results**: A total of 642 postpartum people were randomized. Compared to usual care (*n* = 321), participants randomized to B2H (*n* = 321) reported significantly fewer symptoms of stress (β –4.05, 95% CI –4.99, –3.11), depression (β –1.42, 95% CI –1.94, –0.89), and anxiety (β –0.92, 95% CI –1.52, –0.33) (Figure). They also reported better global health (β 2.69, 95% CI 1.19, 4.19), higher relationship satisfaction (β 2.20, 95% CI 1.03, 3.36), and greater self‐efficacy (β 1.31, 95% CI 0.29, 2.33).

**Conclusion**: B2H led to significant and sustained improvements in mental health, relationship health, and self‐efficacy over the full first year postpartum. These findings underscore the public health potential of scalable, evidence‐based digital tools to address rising rates of perinatal mental health conditions and to improve outcomes for new families.

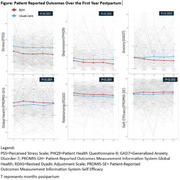

**8:15 AM – 8:30 AM**

## 2 | **Glycemic Measures in Early Pregnancy and Neonatal Outcomes**

Noelia Zork^1^; Alan Kuang^2^; Patrick M. Catalano^3^; Francesca Facco^4^; Maisa Feghali^5^; William A. Grobman^6^; Erin S. LeBlanc^7^; William L. Lowe^8^; Audrey Merriam^9^; Mirella Mourad^10^; Caryn E.S. Oshiro^11^; Camille E. Powe^12^; Uma M. Reddy^1^; Dwight J. Rouse^13^; Jennifer Sherr^14^; Alexandra C. Spadola^15^; Kimberly K. Vesco^7^; Lynn M. Yee^16^; Denise Scholtens^16^; Erika F. Werner^15^

^1^Columbia University Irving Medical Center, New York, NY; ^2^Northwestern University, Chicago, IL; ^3^MGH REU, Boston, MA; ^4^UPMC, UPMC, PA; ^5^Magee Women's Research Institute, University of Pittsburgh, Pittsburgh, PA; ^6^Warren Alpert Medical School of Brown University, Providence, RI; ^7^Kaiser Permanente Center for Health Research, Portland, OR; ^8^Northwestern University, Northwestern University, IL; ^9^Yale School of Medicine, Yale School of Medicine, CT; ^10^Division of Maternal‐Fetal Medicine, Department of Obstetrics and Gynecology, Columbia University Irving Medical Center, New York, NY; ^11^Kaiser Permanente Hawaii, Center for Integrated Health Care Research, Honolulu, HI; ^12^Massachusetts General Hospital, Boston, MA; ^13^Women & Infants Hospital of Rhode Island / Warren Alpert Medical School of Brown University, Providence, RI; ^14^Yale School of Medicine, New Haven, CT; ^15^Tufts University School of Medicine, Boston, MA; ^16^Northwestern University Feinberg School of Medicine, Chicago, IL

**Objective**: To evaluate whether maternal glycemia in early pregnancy is independently associated with adverse neonatal outcomes in individuals without overt diabetes.

**Study Design**: At 9 U.S. sites we conducted a planned secondary analysis of a prospective cohort study of participants aged ≥18 years with singleton gestations who underwent masked 75‐g OGTT, HbA1c, and 10‐days of continuous glucose monitoring (CGM) at 10–14 wks. Births at ≥20 wks without major anomalies were analyzed. The primary outcome was a composite which included neonatal hypoglycemia, respiratory morbidity, birth injury, hyperbilirubinemia, stillbirth, and neonatal death. Exposures variables were OGTT values continuous and binary (any fasting values >101 mg/dL, 1‐h >135 mg/dL, or 2‐h >132 mg/dL), HbA1c (continuous and binary: ≥5.6%), and nocturnal (12 am–6 am) hyperglycemia on CGM (continuous: % time >133 mg/dL; binary: ≥9% time above >133 mg/dL). OGTT, HbA1c, and CGM thresholds were selected to optimize prediction of gestational diabetes in the primary analysis. Generalized linear regression models were sequentially adjusted for pre‐defined sets of potential confounders: Model 1 included participant demographics, Model 2 added socioeconomic factors, and Model 3 added BMI.

**Results**: Among 2037 participants, 239 (11.7%) newborns experienced the composite outcome (Table 1). Fasting glucose (mean 86.8mg/dL, +/− 7.2) was associated with the composite outcome in all three models, though effect sizes were slightly attenuated after adjustment for BMI (RR 1.2, fasting glucose higher by 1SD [95% CI 1.02, 1.30]). The binary OGTT and continuous 1‐h, and 2‐h glucose values were associated with the composite outcome in Models 1 and 2, but not Model 3. HbA1c showed no association with outcomes. Nocturnal hyperglycemia using a binary CGM threshold was associated with outcomes in all three models (RR 1.4 [1.0, 1.9]), whereas continuous CGM was not (Table 2).

**Conclusion**: In this cohort without overt diabetes, fasting glucose and nocturnal hyperglycemia on CGM in early pregnancy were associated with a higher risk for adverse neonatal outcomes.

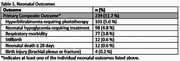

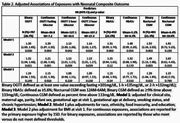

**8:30 AM – 8:45 AM**

## 3 | **Randomized Trial of Bovine Lactoferrin for Treatment of Iron Deficiency in Pregnancy**

Joanne M. Said^1^; Rachel L. O'Connell^2^; Emily Callander^3^; Reynolds M. Rebecca^4^; Michael Dibley^3^; Sajid Soofi^5^; Sidrah Nausheen^5^; Karen M. Baker^6^; Amanda Henry^7^; Raiyomand Dalal^8^; Josephine Laurie^9^; Stephen Cole^10^; Lourdes St George^11^; Amanda Dennis^12^; Christos Georgiou^13^; Irena Idel^14^; Jonathan M. Morris^15^; Valeria Lanzarone^16^; Ben W. Mol^17^; William Tarnow‐Mordi^2^

^1^Western Health / University of Melbourne, University of Melbourne, Victoria; ^2^NHMRC Clinical Trials Centre, University of Sydney, Camperdown, New South Wales; ^3^University of Technology Sydney, Sydney, New South Wales; ^4^University of Edinburgh, Edinburgh, Scotland; ^5^Aga Khan University Hospital, Aga Khan University Hospital, Sindh; ^6^Royal Brisbane and Women's Hospital, Royal Brisbane and Women's Hospital, Queensland; ^7^The George Institute for Global Health, UNSW, Sydney, New South Wales; ^8^Campbelltown Hospital, Campbelltown, New South Wales; ^9^Mater Misericordiae, Brisbane, Queensland; ^10^Epworth Healthcare, East Melbourne, Victoria; ^11^Auburn Hospital, Auburn, New South Wales; ^12^Launceston General Hospital, Launceston General Hospital, Tasmania; ^13^Angliss Hospital, Upper Ferntree Gully, Victoria; ^14^Box Hill Hospital, Box Hill, Victoria; ^15^Royal North Shore, Sydney, New South Wales; ^16^Nepean Hospital, Penrith, New South Wales; ^17^Monash Health, Monash University, Victoria

**Objective**: Iron deficiency anemia affects up to 20% of pregnant women globally with significant adverse maternal and fetal outcomes. Meta‐analyses of randomized trials report that oral bovine lactoferrin (bLF) is as effective as oral ferrous sulfate (FeSO_4_), with fewer gastrointestinal effects. However, the methodological quality of several included trials was considered to be low.

**Study Design**: This was an international, multicentre, double blind randomized trial of the efficacy of bLF versus FeSO_4_ in treating iron deficiency in pregnancy. Institutional ethics approval was obtained and all participants provided written informed consent.

Pregnant women were eligible if they had mild/moderate iron deficiency anemia (Hb ≤110 g/L) OR were iron deficient and likely to become anemic (Hb ≤115 g/L AND serum ferritin ≤20 µg/L). Participants were randomised to *either* 200 mg bLF or FeSO_4_ 80 mg daily.

The primary maternal endpoint was the proportion of women receiving intravenous iron (IVFe) infusion according to local protocol or being anemic (Hb <110 g/L) at any time between 34 weeks’ gestation and delivery. The primary neonatal end point was birth weight by gestational age. Analysis was according to intention to treat. Trial registration ACTRN12614000988651

**Results**: From September 2015 to October 2021, we randomized 919 participants at 15 sites in Australia and Pakistan (460 bLF; 459 to FeSO_4_). Baseline characteristics were comparable (Table 1).

Participants allocated to bLF were more likely to require IVFe infusion or to be anemic (Hb <110 g/L) at any time between 34 weeks and delivery (57% versus 38%; RR 1.50 (95% CI 1.27‐1.76), *p* < 0.0001). There was no difference in neonatal birth weight or rates of birth weight below 10^th^ centile for gestation (Table 2).

There were no differences in reported side effects.

**Conclusion**: In contrast to earlier findings, our trial showed clear superiority of FeSO_4_ over bLF. These results raise concerns about the effectiveness of the bLF product used and suggest FeSO_4_ to be the treatment of first choice.

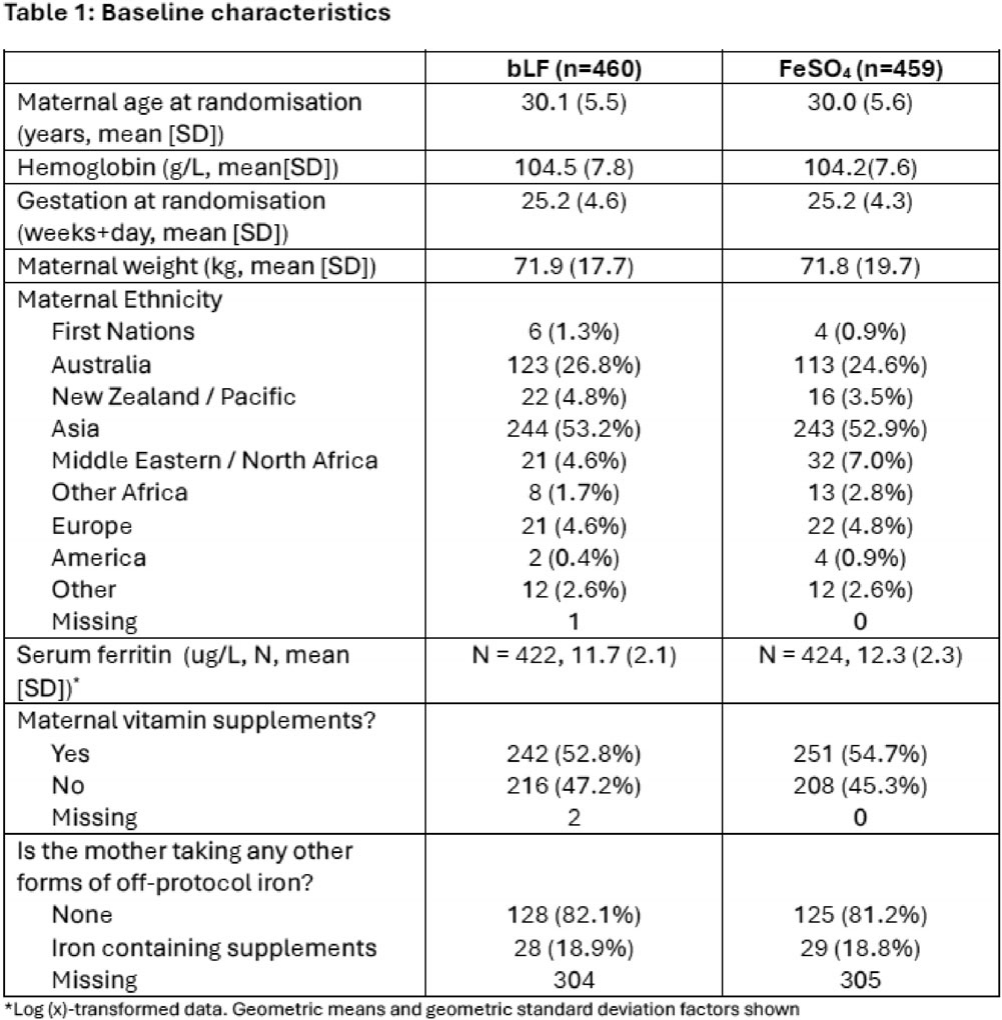

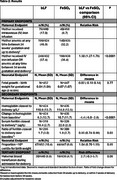

**8:45 AM – 9:00 AM**

## 4 | **Performance of a** Single **Gene Non‐Invasive Prenatal Screening Test for Recessive Conditions**

Rajeevi Madankumar^1^; Thomas Westover^2^; Luis D. Pacheco^3^; Mollie McDonnold^4^; Jessica Scholl^5^; Lawrence D. Platt^6^; Ravindu P. Gunatilake^7^; Olaide Ashimi Balogun^8^; Angela T. Bianco^9^; Laura G. Hendon^10^; Todd Rosen^11^; Ashley S. Roman^12^; Karen Fitch^13^; Karin Niemeyer^13^; Ravi Vijaya Satya^13^; Ebad Ahmed^13^; Xingwu Lu^13^; Wenbo Xu^13^; Hyunseok P. Kang^13^; Fergal Casey^13^; Matthew Rabinowitz^13^; Emily Morton^13^; Vivienne Souter^13^; Jeffrey T. Meltzer^13^; John Williams, III^14^

^1^Northwell Health, New Hyde Park, NY; ^2^Capital Health, Pennington, NJ; ^3^University of Texas Medical Branch, Galveston, TX; ^4^St. David's Women's Center of Texas, Austin, TX; ^5^Weill Cornell Medicine, New York, NY; ^6^UCLA David Geffen School of Medicine and Cedars‐Sinai Medical Center, Los Angeles, CA; ^7^Valley Perinatal Services, Chandler, AZ; ^8^Obstetrix Maternal‐Fetal Medicine Specialist of Houston, Houston, TX; ^9^Icahn School of Medicine at Mount Sinai, New York, NY; ^10^University of Mississippi Medical Center, Jackson, MS; ^11^Rutgers Robert Wood Johnson Medical School, New Brunswick, NJ; ^12^Division of Maternal‐Fetal Medicine, Department of Obstetrics and Gynecology, NYU Grossman School of Medicine, New York, NY; ^13^Natera, Inc., Austin, TX; ^14^Cedars‐Sinai Medical Center, Los Angeles, CA

**Objective**: To report the first milestone readout of the EXPAND study, an ongoing prospective multicenter study evaluating a novel single gene non‐invasive prenatal screening test (sgNIPT). The test uses LinkedSNP™ technology to assess autosomal recessive conditions: cystic fibrosis (*CFTR*), spinal muscular atrophy (*SMN1*), alpha‐thalassemia (*HBA1*/*HBA2*), and beta‐hemoglobinopathies (*HBB*) including sickle cell disease.

**Study Design**: Maternal blood was collected from pregnant carriers of a monogenic condition. Fetal/neonatal variant status was confirmed by clinical diagnostic genetic testing or postnatal buccal swab. Cell‐free DNA from maternal plasma was analyzed using hybrid capture‐based, next‐generation short‐read sequencing. Maternal genomic DNA was analyzed using long‐range PCR to derive the maternal haplotype associated with the variant of interest using common single nucleotide polymorphisms. Relative variant dosage analysis, relative haplotype dosage analysis, and allelic bias correction algorithms assessed fetal risk. Single nucleotide variants, small indels, and copy number variants were evaluated.

**Results**: Test performance was evaluated in 90 pregnant carriers of 101 variants: 44 *CFTR*, 18 *SMN1*, 18 *HBA1/2*, and 21 *HBB* variants. The median maternal age was 31 years (range 20–44). The most common race/ethnicities were White (31%), Black (28%), multiple ethnicities (11%), and Hispanic (7%). The sensitivity for affected fetuses was 91% (10/11) and the PPV was 53% in this study cohort. The sensitivity included 5/5 homozygous fetuses correctly identified, including one fetus affected by homozygous non‐F508del *CFTR* variants. The no‐call rate was 5.9% (6/101).

**Conclusion**: This readout suggests this novel sgNIPT approach can accurately assess fetal risk for recessive conditions from a maternal blood sample. The test successfully detected difficult‐to‐identify homozygous fetuses and variants more common in non‐White ethnicities. While carrier screening of both parents remains the ACOG‐recommended strategy, sgNIPT can be a useful tool when paternal testing is not feasible.

**9:00 AM – 9:15 AM**

## 5 | **A Maneuver to** Decrease **Perineal Lacerations and OASIS: Patient Positioning for Perineal Protection (4P) Study**

Marti D. Soffer^1^; Kaitlyn E. James^2^; Pichliya Liang^3^; Arlin Delgado^2^; Matthew H. Mossayebi^2^; Margaret Hamp^3^; Molly R. Siegel^4^; Mark A. Clapp^5^; Anjali J. Kaimal^6^; William H. Barth, Jr.^1^

^1^Department of Obstetrics and Gynecology, MassGeneral Brigham; Division of Maternal‐Fetal Medicine; Harvard Medical School, Massachusetts General Hospital, Boston, MA; ^2^Department of Obstetrics and Gynecology, MassGeneral Brigham; Division of Maternal‐Fetal Medicine; Harvard Medical School, Boston, MA; ^3^Department of Obstetrics and Gynecology, MassGeneral Brigham, Massachusetts General Hospital, Boston, MA; ^4^Massachusetts General Hospital, Division of Maternal Fetal Medicine, Boston, MA; ^5^Department of Obstetrics and Gynecology, Mass General Brigham, Massachusetts General Hospital, MA; ^6^Department of Obstetrics and Gynecology, University of South Florida, Tampa, FL

**Objective**: To determine the effect of hip extension vs usual positioning at the time of crowning on perineal lacerations and obstetrical anal sphincter injury (OASIS).

**Study Design**: We conducted a non‐blinded RCT of term nulliparous patients with vertex singletons planning vaginal delivery. Once fully dilated, patients were randomized 1:1 to usual care (undirected positioning, typically with hips flexed) or to the intervention (directed hip extension) at crowning/delivery of the head; patients delivered via cesarean were excluded. The primary outcome was significant perineal laceration (SPL), defined as 2^nd^, 3^rd^, or 4^th^ degree lacerations. As a prespecified secondary outcome, OASIS was compared between groups. To detect a 10% reduction in SPL, a sample size of 1206 was needed. Information was collected regarding adherence to the assigned group at delivery. Analysis by intention‐to‐treat and as‐treated was planned a priori.

**Results**: 1828 patients were enrolled from 4/2021 to 7/2025. 1357 were randomized and 1207 delivered vaginally: 612 in the usual care arm, 595 in the intervention arm. By intention‐to‐treat, SPL occurred in 66.3% of the usual care and 66.4% of the intervention group (*p* = 0.99) and OASIS occurred in 8.0% and 8.2% respectively (*p* = 0.88) (Table 1). 25.4% of those randomized to the intervention did not deliver with hips extended; longer 2nd stage and operative vaginal delivery were associated with decreased likelihood of receiving the intervention. As‐treated analyses demonstrated that the intervention reduced the risk of SPL by 10% (69% vs 62%, *p* = 0.02, RR 0.90 [0.83, 0.98]) and OASIS by 47% (9.9% vs 5.3%, *p* = 0.004, RR 0.53 [0.34, 0.83]) (Table 2). The effect on SPL and OASIS remained after adjustment for birthweight, episiotomy, and time in the 2nd stage.

**Conclusion**: Extension of the hips at the time of delivery did not reduce the rates of SPL in the primary analysis; however, 25% assigned to the intervention did not deliver with hips extended. In pre‐specified as‐treated analysis adjusted for confounders, extension of the hips at the time of delivery was associated with reduced SPL and OASIS.

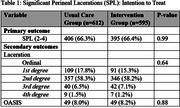

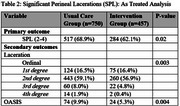

**9:15 AM – 9:30 AM**

## 6 | **Fetal Growth** Restriction**: Comparison of Two National Surveillance Guidelines (FAME)**

Aaron W. Roberts^1^; Mar Romero‐Lopez^2^; John W. Hotra^2^; Eleazar E. Soto^3^; Angela Glaser^4^; Robyn Garcia^2^; Jennie Coselli^2^; Suneet P. Chauhan^5^; Hector Mendez‐Figueroa^6^

^1^Department of Obstetrics, Gynecology and Reproductive Sciences, McGovern Medical School at UTHealth Houston, Houston, TX; ^2^McGovern Medical School at UTHealth, Houston, TX; ^3^University of Texas Health Science Center in Houston, McGovern Medical School, Houston, TX; ^4^UT Houston, Houston, TX; ^5^Delaware Center for Maternal‐Fetal Medicine of Christiana Care, Newark, DE; ^6^Division of Maternal‐Fetal Medicine, Department of Obstetrics, Gynecology and Reproductive Sciences, McGovern Medical School at UTHealth Houston, Houston, TX

**Objective**: SMFM and ISUOG recommend antenatal surveillance (ANS) for fetal growth restriction (FGR), albeit with differing management algorithms. Our primary objective was to compare a composite neonatal adverse outcome (CNAO) among FGR utilizing SMFM vs. ISUOG‐recommended ANS. The secondary objective was to compare composite maternal adverse outcomes (CMAO).

**Study Design**: Our multi‐center pre‐ and post‐intervention study (NCT05938829), conducted over 2.5 years, included singleton non‐anomalous FGR (EFW/AC <10% for GA using Hadlock et al curve). The first group was managed via SMFM‐recommended ANS (EFW, NST, and UA S/D ratio).  The second group was managed according to ISUOG ANS (EFW, NST, UA PI index, MCA, and DV Dopplers).  Delivery timing guidelines were congruent with recommendations from the evaluated ANS.  Using Bayesian statistics, a sample size of 550 in each arm was estimated to detect a 1/3 decrease in the baseline 5% rate for CNAO, with >65% probability of any risk difference (>80% power; possible effect sizes from 1000 simulated posterior distributions). Posterior probability of risk reduction (PPRR), posterior relative risk (pRR), 95% credibility intervals (CrI), and number needed to treat (NNT), or harm (NNH) were calculated.

**Results**: Between the groups, maternal characteristics differed by race and ethnicity but otherwise were similar. Most deliveries occurred ≥34 weeks (88.4% SMFM, 91.5% ISUOG). (Table 1). The primary outcome occurred in 38 of 689 (5.5%) individuals during the SMFM guideline and 45 of 683 (6.6%) with ISUOG, resulting in a 27% PPRR for CNAO in the ISUOG group (pRR 1.19, 95% CrI 0.76–1.83). Compared to SMFM, ISUOG guidelines produced a NNH of 91 of increasing CNAO (95% CI 71–125). CMAO occurred in 9.1% during SMFM and 8.3% in ISUOG, a 73% PPRR (pRR 0.90, 95% CrI 0.61–1.31). The NNT to reduce CMAO was 125 (95% CI 80–279; Table 2).

**Conclusion**: Among a diverse, low and high‐risk FGR cohort, the use of ISUOG guidelines failed to decrease adverse neonatal outcomes compared to SMFM (6.6% vs 5.5%), with 73% posterior probability of harm.

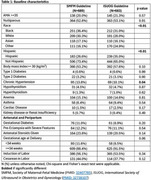

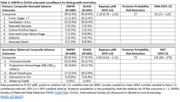

**9:30 AM – 9:45 AM**

## 7 | **Randomized Trial of Cervical Pessary or Progesterone vs Placebo for Short Cervix in Twin Gestations**

Joseph R. Biggio, Jr.

On behalf of the Eunice Kennedy Shriver National Institute of Child Health and Human Development (NICHD) Maternal‐Fetal Medicine Units (MFMU) Network, Bethesda, MD

Ochsner Health, New Orleans, LA

**Objective**: Over half of twin gestations deliver preterm. Randomized trials in unselected twin gestations have not demonstrated that vaginal progesterone (VP) or Arabin cervical pessary (AP) reduce preterm birth (PTB), but in the subgroup of patients with a short cervix some have noted benefit. Our objective was to evaluate whether VP or AP reduce the frequency of PTB in individuals with twin gestations and a short cervix.

**Study Design**: Three‐group randomized, multicenter trial comparing AP or VP 200mg daily with placebo in twin gestations with a transvaginal cervical length (CL) of ≤30 mm at 16 wk 0 d‐23 wk 6 d. Study personnel performing pessary placement underwent training and passed a certification test. Monoamnionicity, fetal anomaly, and twin‐twin transfusion syndrome were exclusions. Our primary outcome was delivery or fetal death of either twin prior to 35 weeks; planned sample size was 630 to detect a 1/3 reduction in the placebo group primary outcome rate of 55% with 90% power.

**Results**: From 2015–2024, 7484 patients had CL screening and 1551 with CL ≤30 mm were identified (20.7%). Due to recruitment difficulties related to changing clinical practice and funding termination, the study was halted after enrolling 437 patients. Baseline characteristics were balanced between groups, including median CL and gestational age (GA) (Table). In intent‐to‐treat analysis, the primary outcome occurred in 57.5% of the VP group, 55.3% of the AP group, and 63.2% of the placebo group: RR 0.91, 95% CI 0.73–1.13 for VP vs. placebo; RR 0.87, 95% CI 0.70–1.09 for AP vs. placebo. GA at delivery and the proportion of participants undelivered by week of gestation did not differ among groups (Figure). There was no difference in perinatal death for either VP (RR 0.69, 95% CI 0.32, 1.47) or AP (RR 0.71, 95% CI 0.40, 1.27) compared to placebo. Other maternal and neonatal outcomes were similar across groups.

**Conclusion**: Neither placement of a pessary nor treatment with vaginal progesterone reduced the risk of preterm birth or improved neonatal outcomes among individuals with twin gestations and a CL ≤30 mm.

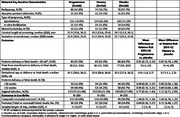

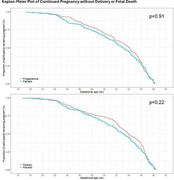

**9:45 AM – 10:00 AM**

## 8 | Association Between SARS‐CoV‐2 Vaccine in Pregnancy and Child Neurodevelopment at 18–30 Months

George R. Saade^1^; Brenna L. Hughes^2^

On behalf of the Eunice Kennedy Shriver National Institute of Child Health and Human Development Maternal‐Fetal Medicine Units Network, Bethesda, MD

^1^Eastern Virginia Medical School at Old Dominion University, Norfolk, VA; ^2^Department of Obstetrics and Gynecology, Duke University School of Medicine, Duke, NC

**Objective**: To evaluate whether there is an association between SARS‐CoV‐2 vaccine in pregnancy and offspring neurodevelopmental measures.

**Study Design**: Multi‐center prospective observational study of 18–30 month old offspring of mothers who did (exposed) or did not (unexposed) receive at least one dose of an mRNA SARS‐CoV‐2 vaccine during or within 30 days prior to pregnancy. Exclusions included delivery <37 weeks, multifetal pregnancy and major congenital malformations. Eligible participants were identified by electronic record review and contacted for consent. Unexposed women were matched with exposed women by delivery site, delivery date, insurance status, and race. The primary outcome was equivalence within an upper and lower margin of 10 points for the Ages and Stages Questionnaire version 3 (ASQ‐3) total score. Secondarily, associations with domain‐specific ASQ‐3 scores as well as Child Behavior Checklist, Modified Checklist for Autism in Toddlers and Early Childhood Behavior Questionnaire scores were evaluated.

**Results**: Of 3956 mothers assessed, 271 exposed and 240 unexposed were enrolled. Matching to unexposed on all characteristics was possible in 217 pairs. The mothers in the exposed group were more likely to be nulliparous, and the children were more likely to be vaccinated and slightly younger at assessment than unexposed (median [IQR] months: 25.4 [24.5, 28.1] vs 25.9 [24.7, 28.5]; *p* = 0.03). There were no other significant differences in baseline characteristics. The primary outcome of ASQ score was equivalent between exposed and unexposed (255 vs 260, median difference ‐3.4, 95% CI: ‐9.7,3.0; Table 1). No significant differences were noted in secondary outcomes (Table 2).

**Conclusion**: The primary neurodevelopmental outcome was equivalent between children of mothers who received and did not receive mRNA SARS‐CoV‐2 vaccination during or immediately prior to pregnancy. There was no association with secondary neurodevelopmental outcomes. Our results provide reassurance regarding the safety of mRNA SARS‐CoV‐2 vaccination in pregnancy.